# Conditioned Pain Modulation, Placebo and Offset Analgesia: Rates of Behavioural Expression of Inhibitory, Nonresponse and Facilitatory Pain Modulatory Effects

**DOI:** 10.1002/ejp.70088

**Published:** 2025-07-22

**Authors:** Lewis S. Crawford, Ashleigh Wake, Rebecca V. Robertson, Allan Peng, Noemi Meylakh, Damien C. Boorman, Leana Sattarov, Alister Ramachandran, Vaughan G. Macefield, Kevin A. Keay, Luke A. Henderson

**Affiliations:** ^1^ School of Medical Sciences (Neuroscience) Brain and Mind Centre, University of Sydney Sydney New South Wales Australia; ^2^ Westmead Hospital Pain Management Centre Sydney New South Wales Australia; ^3^ Department of Neuroscience Monash University Melbourne Victoria Australia

## Abstract

**Background:**

The brain is capable of powerfully inhibiting perceived pain intensity. Experimentally, three pain modulating phenomena have been well explored: placebo analgesia (PA), offset analgesia (OA) and conditioned pain modulation (CPM). While all three can reduce pain intensity, these paradigms are not often compared behaviourally, nor are their potentially common psychological or physiological mechanisms considered.

**Methods:**

Here, we present retrospective behavioural pain rating, psychological and demographic data in 273 pain‐free control participants who underwent either PA (*n* = 100), OA (*n* = 37) or CPM (*n* = 136). Significant changes in pain intensities were assessed using permutation testing to derive cohorts where pain was significantly inhibited (inhibitory responders), unchanged (nonresponder) or increased (facilitatory responder) during the expression of each phenomenon. Psychological questionnaire scores, demography and pain perception variability were compared between response cohorts to all three phenomena.

**Results:**

We identified largely similar proportions of individuals categorised as either inhibitory responders, nonresponders or facilitatory responders to each of PA, OA and CPM—with no sex differences identified in any phenomena nor response category. Dispositional optimism and calibrated noxious temperature demonstrated a significant effect in PA and CPM responses, respectively—with inhibitory responders recording higher scores than facilitatory responders in PA, and inhibitory responders possessing lower thermal sensitivity to nonresponders in CPM.

**Conclusion:**

A shared mechanism was identified between PA and OA, such that perceived pain variability to repeated identical noxious stimuli related to both phenomena's expression. This warrants further investigation given the suggested neural circuit differences between these two phenomena, and only PA is presently linked with Bayesian theorem.

**Significance Statement:**

Humans are capable of inhibiting their own pain in several ways; however, the brain systems driving these analgesic mechanisms are complex and are known to diverge despite producing similar outcomes. Here we show core behavioural similarities between different forms of endogenous analgesic phenomena, with differences in psychological and physiological correlates.

## 1 Introduction

It is widely appreciated that the brain can *modulate* incoming nociceptive sensory signals. As early as around 400 bce, Hippocrates noted in his *Aphorisms* that the painful numbness from cold water exposure could dull visceral pain, essentially describing the conditioned pain modulation phenomena (Chance 1930). Moreover, the observations of Henry Beecher during the Second World War that many soldiers with significant injuries reported pain relief at the hands of sham analgesics gave rise to modern investigations of placebo analgesia (Beecher 1955). More recently, the seminal work of Grill and Coghill defined offset analgesia phenomena, whereby a minor decrease in noxious intensity can produce a large decrease in perceived pain, establishing a triad of pain modulatory mechanisms capable of being elicited in humans without pharmacological intervention (Grill and Coghill 2002). It is now well established that the brain can both inhibit and enhance the intensity of perceived pain and that an individual's endogenous pain modulatory ability is associated with the presence of chronic pain (Gagné et al. 2020; Hashmi et al. 2012; Pickering et al. 2014; Schmid et al. 2015; Szikszay et al. 2020) and with the ability to respond to particular chronic pain treatments (Dürsteler et al. 2021; Kono et al. 2023).

Since their discovery, these three analgesic phenomena: placebo analgesia (PA), conditioned pain modulation (CPM) and offset analgesia (OA), have been routinely used to evoke endogenous pain modulatory mechanisms in humans. PA is characterised by a pain intensity reduction due to the expectation of pain relief even though the applied treatment is inert. Previous investigations have demonstrated this effect to be largely driven by expectancy modulation, conditioning effects or a combination of the two (Amanzio and Benedetti 1999). To some degree this effect is opiate driven, since naloxone administration can partially or entirely abolish the behavioural effects of PA (Benedetti 1996; Grevert et al. 1983; Zhang et al. 2013). Regardless of the mechanism that underpins PA, it has been recently proposed that PA follows Bayesian principles, with prior experiences with administered placebo substances forming experimental priors, of which future likelihoods are shifted towards to produce the behavioural posterior of reduced pain (Büchel et al. 2014; Grahl et al. 2018).

CPM is characterised by a pain intensity reduction during noxious stimulation in one body location when a second noxious stimulus is concurrently applied to a distant body location. This phenomenon is the human homologue of the diffuse noxious inhibitory control (DNIC) reaction first observed by le Bars et al. (1979) and later translated to humans by the same research team (le Bars et al. 1992). Largely understood to follow the theorem of gate control, CPM expression relates to spatial filtering of excess noxious information and can be abolished in experimental rodents by chemical ablation of brainstem nuclei (Villanueva et al. 1996).

OA is characterised by a disproportionally large decrease in pain intensity during a minor reduction in noxious stimulus intensity. Unlike CPM, OA involves temporal filtering of noxious information, suggesting a time‐locked effect of noxious processing such that when an individual is exposed to a minor increase in intensity, swift re‐exposure to an initially moderate intensity will induce a larger decrease in perceived pain (Yelle et al. 2009). Despite being seemingly sensory‐driven in nature, OA expression can be manipulated by altering expectancy (Szikszay et al. 2023), and is associated with activation change in higher‐order cortical sites associated with cognition (Alter et al. 2022). It is known that not all individuals display a significant pain decrease during these three paradigms (Amanzio and Benedetti 1999; Szikszay et al. 2019; Youssef et al. 2016b) and that these variations in responsiveness relate to functional changes in neural circuitry—suggesting their expression ties directly to neurobiological individual differences (Christensen et al. 2020; Crawford et al. 2021; Darragh et al. 2015; Wang et al. 2022; Youssef et al. 2016a, 2016b).

Interestingly, despite all three of these phenomena intending a similar output, that is, a nonpharmacologically driven change in perceived pain, it has been established that they rely on dissimilar mechanisms to achieve this. In the case of CPM and OA, a within‐subject investigation by Nahman‐Averbuch et al. (2014) reported that brain activation patterns do not overlap between phenomena. Furthermore, Niesters et al. (2011) demonstrated unique pharmacological properties of CPM and OA, with CPM and not OA expression blocked by ketamine infusion. This same work showed that ketamine infusion actually *increased* pain in a CPM model—demonstrating the flexibility of pain perception under pain modulatory paradigms, which can fluctuate between analgesic or hyperalgesic expression.

Indeed, it is now appreciated that these ‘analgesic’ paradigms can also evoke pain increases in some individuals, with previous studies reporting pain increases and decreases during CPM (Firouzian et al. 2020; Graeff et al. 2021; Harper et al. 2018; Schliessbach et al. 2019), OA (Cosentino et al. 2025) and PA (Crawford et al. 2021). It is possible that different brain circuits underpin the increases compared to decreases in pain intensity expressed during these pain modulatory paradigms, and thus grouping all individuals together would not be an appropriate way to explore these different responses.

The aim of this retrospective investigation was to determine the proportions of individuals within three independent sample groups that display an inhibitory (pain reduction), facilitatory (pain increase) or no significant (nonresponder) change in pain intensity during PA, CPM and OA paradigms. We hypothesise that, as appears to be the case for placebo, in each sample group approximately 25% of individuals will display a significant increase in pain intensity during these three analgesic paradigms. Furthermore, we aim to determine the relationships between stimulus intensity, pain rating variability, age, sex and psychological measures such as pain catastrophising and anxiety on the response profiles. We hypothesise that greater pain intensity variability and anxiety levels will be associated with a greater chance of expressing a pain increase during each of the three paradigms.

## 2 Methods

### 2.1 Ethics

All experimental procedures were approved by the University of Sydney Human Research Ethics Committee and satisfied the Declaration of Helsinki, with the exception of registration in a database. Written informed consent was obtained from participants at the commencement of the study. At the conclusion of testing, participants were informed both verbally and through a written statement of the necessary deception and true methodology of the experiment and were invited to seek clarification of what they had just experienced.

### 2.2 Participants

A total of 273 healthy control participants were recruited for the study. One hundred participants completed the placebo protocol (45 male, 55 female; mean ± SEM age, 23.40 ± 0.40 years), 136 participants completed the CPM protocol (72 male, 64 female; mean ± SEM age, 32.19 ± 1.19 years), and 37 participants completed the offset protocol (15 male, 22 female; mean ± SEM age, 25.16 ± 1.05 years). Before beginning the study, participants completed a data sheet recording current medication(s) and any alcohol or caffeine ingested in the 24 h prior to testing. The data presented here is a conglomeration of a number of studies whose aim is to define pain modulatory circuits using functional magnetic resonance imaging. All or part of the arm placebo analgesia data has been previously published as part of a brain imaging investigations (Crawford, Meylakh, et al. 2023; Crawford et al. 2021; Crawford, Mills, et al. 2023). It should be noted that all pain rating data presented here was collected from individuals whilst they lay inside a magnetic resonance imaging scanner.

## 3 Experimental Design

Prior to starting the experimental procedure, each participant was asked to complete a series of questionnaires that consisted of (i) The Revised Life Orientation Test (LOT‐R): developed to assess individual differences in generalised optimism versus pessimism (Scheier et al. 1994); (ii) The State–Trait Anxiety Inventory (STAI): developed to measure trait and state anxiety (Spielberger et al. 1971); (iii) pain catastrophising questionnaire (PCS): developed to assess catastrophic thinking related to pain (Sullivan 1995); and (iv) Behavioural Activation and Behavioural Inhibition Scales (BAI): developed to assess two general motivational systems—behavioural inhibition system (BIS) and a behavioural activation system (BAS) (Carver and White 1994). A summary of psychological constructs measured by these questionnaires and their relative scales and limits is provided in Table S1. All of the 100 participants that completed the placebo protocol and 36 of the 37 participants that completed the offset protocol completed all five questionnaires. Of the 136 CPM participants, only 50 completed the LOT and BAI, 109 completed the STAI, and 104 completed the PCS questionnaires.

### 3.1 Placebo

**FIGURE 1:**
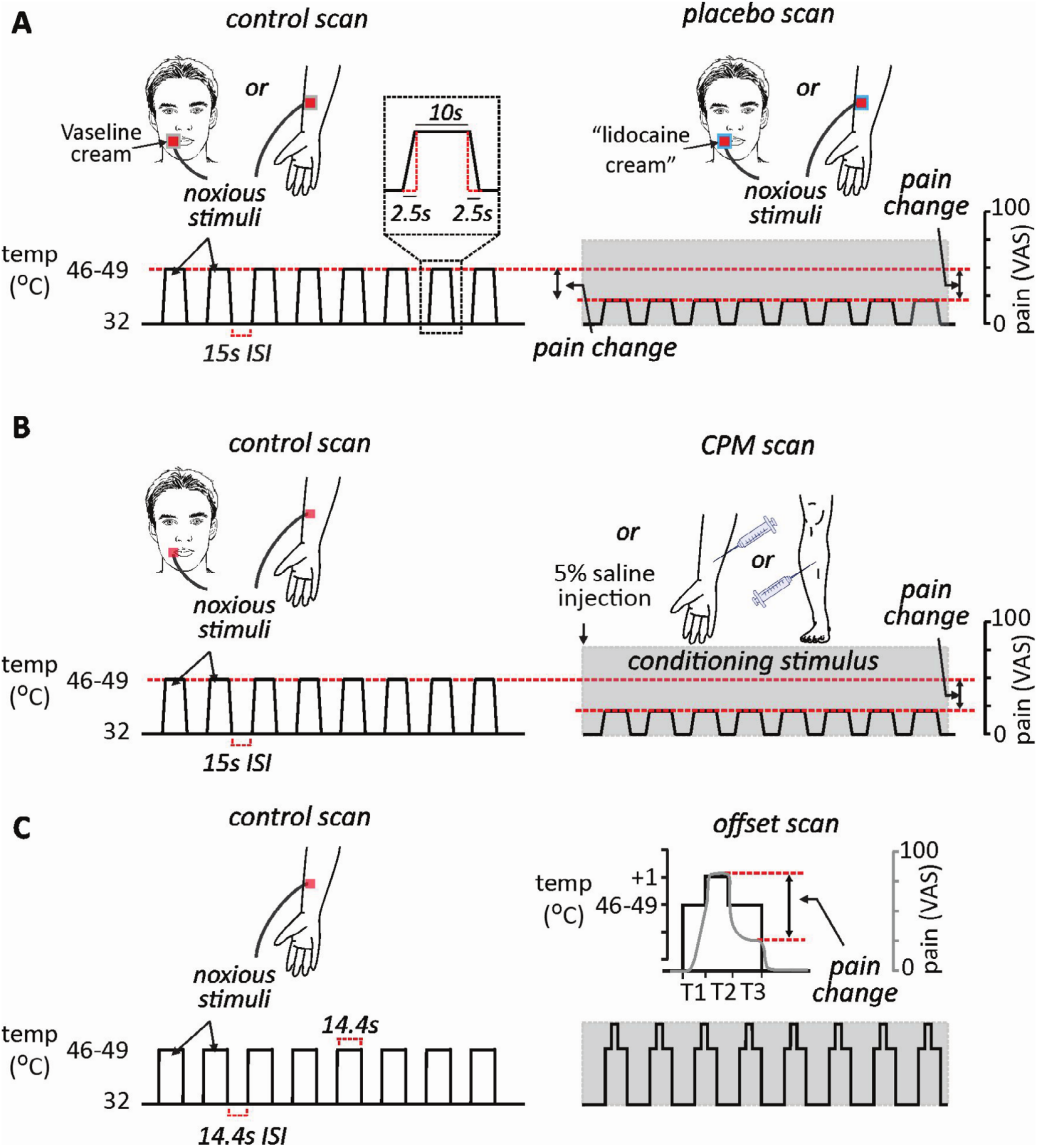
(A) Placebo pain modulation protocol. A series of eight noxious thermal stimuli predetermined to elicit a moderate pain intensity, were delivered to either the right side of the face or right forearm on a Vaseline control cream. During a subsequent scan, the same noxious stimuli were delivered to an immediately adjacent site covered by a ‘lidocaine’ cream that participants were conditioned to believe had pain relieving qualities. Each noxious stimulus lasted 15 s including a 2.5 s ramp up and down and there was an inter‐stimulus intervale (ISI) of 15 s. The change in pain intensity assessed using an on‐line 100‐point visual analogue scale (VAS), during the control and placebo scans were used to determine whether pain had significantly decreased, increased or not changed. (B) Conditioned pain modulation (CPM) protocol. A series of eight noxious stimuli, identical to those delivered during the placebo paradigm was delivered to either the right side of the face or right forearm. Following this series, a 0.5 mL bolus injection of 5% saline was made into either the forearm or lower leg to produce a sustained muscle pain of moderate intensity. A series of eight noxious stimuli were again applied to the right side of the face or forearm during the muscle pain stimulus. The change in pain intensity assessed using an on‐line 100‐point visual analogue scale (VAS), during the control and CPM scans were used to determine whether pain had significantly decreased, increased or not changed. (C) Offset pain modulation protocol. A series of eight noxious stimuli were applied to the right forearm. The ramp up and down period was essentially instantaneous and each stimulus period lasted 14.4 s with an ISI of 14.4 s. During the second series another eight noxious thermal stimuli were delivered (T1), however after 4 s the stimulus temperature was increased by 1°C for 4 s (T2) and then decreased by 1°C for 6.4 s (T3) before returning to baseline. Change in pain intensity was assessed using an online 100‐point visual analogue scale (VAS), during the T3 period in the offset series, and the last 6.4 s of each stimulus block in the control series, and were used to determine whether pain had significantly decreased, increased or not changed.

This study was performed on 23 individuals for face placebo (14 females) and 77 individuals for arm placebo (41 females) and included three sessions occurring on two successive days: a conditioning session on Day 1, and a reinforcement and test session on Day 2 (Figure 1A). Throughout the study, noxious stimuli were administered to either the volar surfaces of participants' left and right forearms or the left and right face using a 3 × 3 cm MR‐compatible Peltier element thermode, which delivered a heat stimulus at a preprogrammed temperature via a Thermal Sensory Analyser (TSA‐II) (Medoc, Israel). Each stimulus lasted 15 s, including a ramp‐up period (4°C/s), a plateau period at a noxious temperature and a ramp‐down period (4°C/s). Each stimulus was separated by a 15 s interstimulus interval (ISI) at a nonpainful temperature of 32°C. Throughout conditioning, participants rated their pain online using a horizontal 10 cm visual analogue scale (VAS), ranging between 0 and 100, where 0 was described as ‘no pain’ and 100 as ‘the worst pain imaginable’. During testing for placebo analgesia, participants used an MR‐compatible two‐button Cedrus lumina button box to continuously report their perceived pain. Participants controlled the position of a slider to report their pain, holding the left (moved slider towards zero) or right (moved slider towards 100) button with their left middle and index finger, recording a trace of perceived pain continuously throughout the two sequences.

#### 3.1.1 Conditioning

Session 1 consisted of two rounds of a response conditioning protocol. Participants were first informed both verbally and via a written statement that the study was designed to investigate the modulatory effects of a topical pain inhibitor, which had been shown to decrease pain in some individuals. A second control cream was stated to be purely Vaseline and described as being necessary to evaluate typical pain responses. In reality, both creams contained solely Vaseline and only differed in colour and their described properties. Thermal calibration was conducted, where 10 randomised stimuli ranging from 44°C–48.5°C in 0.5°C intervals were delivered to the left forearm or face. Participants were informed that we were interested in recording a temperature that elicited a moderate subjective pain response (40–50 VAS rating) and that this temperature would be used throughout the remainder of the experiment. However, using the ratings provided during this calibration, we recorded two different temperature stimuli: a moderate pain temperature (40–50 VAS rating) and a low pain temperature (20–30 VAS rating). These two temperatures were then deceptively applied to the different cream sites throughout the remainder of Sessions 1 and 2, such that the low‐temperature stimulus was delivered to the site on which the placebo analgesic cream had been applied, to convince the participant that this was indeed a less painful stimulus.

To increase believability that the creams contained active substances, false labels were attached to the cream bottles and green food colouring was added to the ‘analgesic’ cream. Creams were then applied to two adjacent 3 × 3 cm squares on the participants' right forearm or face. Ten minutes following cream application, we conducted two rounds of conditioning. Participants were informed they would be receiving eight identical moderate thermal stimuli to both cream sites and were instructed to report their perceived pain intensity using the VAS. As noted above, during the two conditioning rounds we deceptively applied a moderate temperature to the control Vaseline site and a low temperature to the placebo lidocaine site.

#### 3.1.2 Reinforcement and Test

At approximately the same time on the following day, Sessions 2 and 3 were conducted with participants inside an MRI scanner and consisted of a reinforcement protocol (Session 2) and a test protocol (Session 3). The creams were reapplied to the forearm or face, in the same order and locations as Session 1, and participants were reminded of the ‘analgesic’ cream's pain‐decreasing qualities. To ensure that the protocol for conditioning was consistent between subsequent days, despite the change in immediate environment (now inside an MRI), reinforcement was conducted by applying four noxious stimuli at the same moderate and low temperatures used throughout Session 1. This was performed on the opposite forearm or face to prevent sensitisation of the testing area.

Following reinforcement, we waited 15 min for residual pain and sensitivity to dissipate. Unlike the conditioning and reinforcement phases, during the test phase we applied *identical moderate temperature stimuli* to both the control Vaseline‐ and placebo analgesic sites (Figure 1A). We asked each participant to report their pain intensity continuously throughout the duration of the scan using the button box and digital VAS. VAS responses were recorded every 0.5 s, and values during each pain period were averaged, providing a pain intensity for each noxious stimulus period. Each participant received two consecutive series of eight stimuli, with the control Vaseline site stimulated first followed by the placebo analgesic site.

### 3.2 CPM

This study was performed on 107 individuals for face CPM (47 females) and 29 individuals for arm CPM (17 females). Participants had the 3 cm × 3 cm MRI‐compatible thermode placed on either their left forearm or left face, and an identical calibration phase as described in the placebo protocol was performed. The temperature that elicited a 5/10 pain intensity rating was used for the subsequent experiment. With the participant inside the MRI scanner, the thermode was placed onto the right side of the arm or face, and a small needle attached to a 1 mL syringe filled with 5% saline was inserted into either the flexor carpi radialis muscle in the right forearm for face CPM, or into the tibialis anterior muscle of the right leg for arm CPM.

**FIGURE 2:**
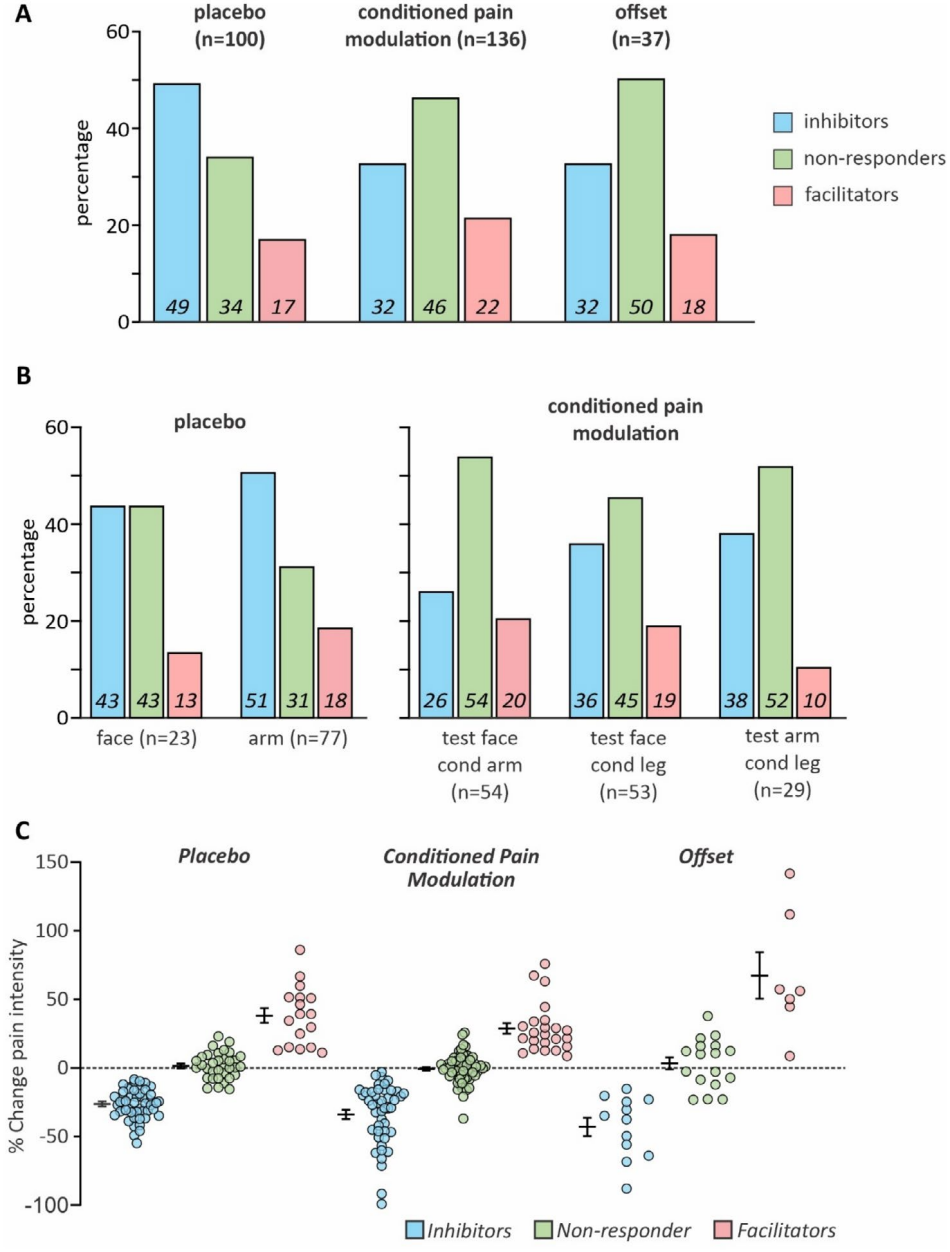
(A) Plots of proportions of individuals that display significant pain intensity decreases (inhibitors), increases (facilitators) or no change (nonresponders) during placebo, conditioned pain modulation and offset paradigms. Numbers at the bottom of each bar indicate the percentages. (B) Plots of proportions of inhibitors, facilitators or nonresponders during placebo applied to the face or arm and conditioned pain modulation where the noxious thermal stimuli are applied to either the face or arm and the conditioning muscle pain stimulus to the arm or leg. Numbers at the bottom of each bar indicate the percentages. (C) Plots of individual and mean ± SEM percent pain intensity changes in inhibitors, nonresponders and facilitators during placebo, conditioned pain modulation and offset paradigms.

CPM was assessed over two separate functional MRI recordings. During the first recording, a series of thermal stimuli with an identical temporal profile to that described for the placebo study were delivered via the thermode, using the temperature calibrated to elicit a moderate pain intensity (test stimulus). This was repeated for a total delivery of eight noxious thermal stimulation periods (Figure 2A). Participants continuously rated their pain on a 10 cm line using an MR‐compatible button box. Immediately prior to a second recording, 0.5 mL of 5% hypertonic saline was injected into either the right forearm or leg to induce sustained muscle pain (conditioning stimulus). Concurrently, eight noxious thermal stimulations were delivered, identical to those delivered in the control scan. VAS responses were recorded at the same rate as described in placebo, averaged to each stimulus period in either recording. At the conclusion of the fMRI scan, participants were also asked to provide an average rating of the intensity of the conditioning (saline) stimulus. During the CPM paradigm, participants rated their pain as in the placebo paradigm, with a two‐button Cedrus lumina button box and digital VAS visible inside the MRI scanner.

### 3.3 Offset

**FIGURE 3:**
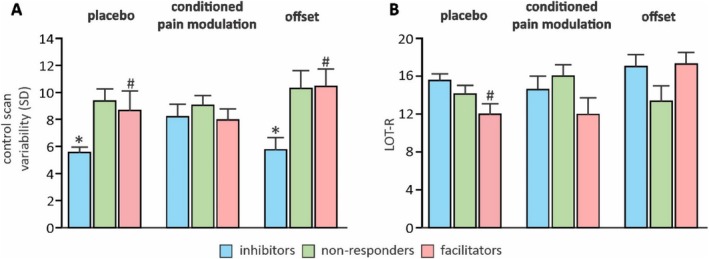
(A) Plots of mean ± SEM pain intensity rating variability (standard deviation: SD) during the initial control series in inhibitor, facilitator and nonresponder groups during placebo, conditioned pain modulation and offset paradigms. (B) Plots of mean ± SEM LOT‐R values for inhibitor, facilitator and nonresponder groups during placebo, conditioned pain modulation and offset paradigms. *Inhibitors significantly different to nonresponders; ^#^inhibitors significantly different to facilitators.

This study was performed on 37 individuals (22 females). Participants had an MRI‐compatible thermode placed onto their left forearm, and the same calibration task was performed in placebo and CPM. Given the requirement for a quicker change in stimulus temperature for this paradigm, we used a different MRI‐compatible thermode capable of a ramp up and down speed of 300°C/s (QST labs, Strasbourg). The temperature that elicited a 5/10 pain intensity rating was used for the subsequent experiment. With the participant inside the MRI scanner, the thermode was placed onto the right forearm. During the first (control) scan, a series of eight noxious thermal stimuli that had an essentially instantaneous ramp‐up period, a plateau period at a noxious temperature of 14.4 s, and an instantaneous ramp‐down period, and an interstimulus period of 14.4 s were delivered using the temperature predetermined as eliciting a moderate pain intensity (test stimulus; Figure 3A). Participants continuously rated their pain using the same MR‐compatible two‐button Cedrus lumina button box with their left middle and index fingers. During a second series, the same eight thermal stimuli were delivered; however, the moderate intensity was held for only the first 4 s of each stimulus period (T1 period). It was then raised by 1°C for 4 s (T2 period), before once again being lowered to the moderate intensity for the remaining 6.4 s (T3 period). VAS responses were recorded at the same rate as described in placebo and CPM, with an average VAS recording calculated specifically during the T3 period of each stimulus block.

### 3.4 Behavioural Classification of Participants as *Inhibitors* (Pain Reduction), nonresponders and *Facilitators* (Pain Increase)

Participants were grouped as either an inhibitor, nonresponder or facilitator based on a bootstrapped permutation procedure (Berry et al. 2011). Briefly, mean VAS ratings to each of the first of the two series described above (i.e., eight moderate intensity thermal stimuli) were entered into a permutation model, where 10,000 artificial samples were generated with replacement. This artificial sample was significance tested against 10,000 artificial samples generated from the VAS ratings to each of the eight noxious stimuli delivered to the ‘analgesic’ placebo cream (placebo analgesia), during the processing of two stimuli concurrently (CPM) or during a minor decrease in noxious intensity (offset analgesia). If the mean difference between the two series was significant, with the ‘analgesic’ placebo, dual stimulus, or offset VAS responses significantly higher than the initial series, a participant was considered a facilitator. If the ‘analgesic’ placebo, dual stimulus or offset VAS responses were significantly lower than the initial series, a participant was considered an inhibitor. If neither condition was satisfied to a standard of *p* < 0.05 one‐tailed distribution, the participant was considered a nonresponder. Examples of facilitator, nonresponder and inhibitor distributions generated from bootstrapped permutation are provided in Figure S1.

We determined if there were significant differences between the proportions of inhibitor, nonresponder and facilitator participants between the placebo, CPM and offset protocols as well as the proportion of females and males in each group (*p* < 0.05, two independent population proportions test). We also determined whether significant differences emerged between the inhibitor, nonresponder and facilitator groups with respect to sex, age, thermode temperature, as well as control and placebo/CPM/offset recording VAS rating variability. Kolmogorov–Smirnov Test of Normality was used to determine whether data were normally distributed and subsequent *t*‐tests were used to determine significant group differences for normally distributed data (*p* < 0.05, Bonferroni corrected for multiple comparisons) and Mann–Whitney U for non‐normally distributed data (*p* < 0.05, Bonferroni corrected for multiple comparisons). Control and placebo/CPM/offset scan pain intensity variabilities were determined by calculating the mean pain intensity during each stimulus period and then calculating the standard deviation of the eight pain intensity values for each of the two scans.

Additionally, for each psychological questionnaire administered and within each phenomenon, single factor ANOVA was performed using scores calculated from inhibitor, nonresponder and facilitator groups. Where significance was identified, two‐sample *t*‐tests were then performed to determine between which two groups' scores significantly differed from each other (*p* < 0.05).

## 4 Results

### 4.1 Group Proportions

Separating individuals in inhibitor, nonresponder and facilitator groups revealed relatively consistent proportions of individuals in each group for each of the three pain modulatory paradigms (Figure 2A). All three paradigms resulted in no significant differences in the proportions of facilitators, nonresponders and inhibitors (*% inhibitors* vs *nonresponders* vs *facilitators*: placebo 48 vs. 34 vs. 17; CPM 32 vs. 46 vs. 22; offset 32 vs. 50 vs. 18). Examination of group proportions during placebo and CPM paradigms administered at different body locations also revealed similar proportions and effect sizes in both a main effect and group level—with large effect sizes noted in inhibitor and facilitator groups of all phenomena irrespective of body site, and predominantly medium effect sizes when considered as the main effect of each phenomenon (Table 1) (*% inhibitors* vs *nonresponders* vs *facilitators*: placebo face 43 vs. 43 vs. 13; placebo arm 51 vs. 31 vs. 18; CPM test face conditioning arm 26 vs. 54 vs. 20; CPM test face conditioning leg 36 vs. 45 vs. 19; CPM test arm conditioning leg 38 vs. 52 vs. 10) (Figure 2B).

**TABLE 1:** Paired effect size estimates, mean and standard deviation differences, *p* values and *t* values during placebo, conditioned pain modulation and offset analgesia paradigms as a main effect and by response classes.

		Cohen's D	Average of difference	Standard deviation of difference	*t*	*p*
*Placebo*						
Main effect	All (*n* = 100)	0.34	−3.69	10.81	−3.42	< 0.001*
	Arm (*n* = 77)	0.31	−3.48	11.26	−2.71	0.008*
	Face (*n* = 23)	0.47	−4.38	9.31	−2.26	0.34
Inhibitors	All (*n* = 49)	2.57	−12.42	4.83	−18.01	< 0001*
	Arm (*n* = 39)	2.58	−12.27	4.75	−16.14	< 0.001*
	Face (*n* = 10)	2.43	−13.02	5.36	−7.68	< 0.001*
Nonresponders	All (*n* = 34)	0.11	0.41	3.75	0.64	0.23
	Arm (*n* = 24)	0.11	0.44	3.98	0.54	0.59
	Face (*n* = 10)	0.10	0.34	3.32	0.33	0.75
Facilitators	All (*n* = 17)	1.90	13.27	6.98	7.84	< 0.001*
	Arm (*n* = 14)	2.05	14.26	6.97	7.65	< 0.001*
	Face (*n* = 3)	1.46	8.65	5.94	2.52	0.13
*CPM*						
Main effect	All (*n* = 135)	0.25	−2.89	11.75	−2.86	0.005*
	Arm (*n* = 29)	0.20	−3.51	8.05	−2.35	0.03*
	Face (*n* = 107)	0.22	−2.72	12.60	−2.23	0.03*
Inhibitors	All (*n* = 45)	1.46	−14.67	10.04	−9.80	< 0.001*
	Arm (*n* = 11)	2.69	−12.14	4.51	−8.94	< 0.001*
	Face (*n* = 34)	1.38	−15.48	11.20	−8.06	< 0.001*
Nonresponders	All (*n* = 68)	0.07	−0.30	4.14	−0.59	0.55
	Arm (*n* = 15)	0.13	0.35	2.60	0.52	0.61
	Face (*n* = 53)	0.11	−0.48	4.49	−0.78	0.44
Facilitators	All (*n* = 23)	1.93	12.50	6.46	9.28	< 0.001*
	Arm (*n* = 3)	2.82	8.81	3.13	4.88	0.04*
	Face (*n* = 19)	1.95	13.06	6.70	8.72	< 0.001*
*Offset*						
Main effect	Arm (*n* = 36)	0.13	−2.30	17.64	−0.78	0.44
Inhibitors	Arm (*n* = 12)	1.98	−21.39	10.79	−6.87	< 0.001*
Nonresponders	Arm (*n* = 17)	0.33	1.83	5.52	1.36	0.19
Facilitators	Arm (*n* = 7)	1.72	20.40	11.89	4.54	0.004*

*Note:* Dark grey boxes and * denote significant differences in perceived pain *p* < 0.05.

We also determined whether there were differences in the proportions of males and females in the inhibitor, nonresponder or facilitator groups for all three paradigms. We found no significant differences in each group for placebo (*females* vs *males*: inhibitors 22 vs. 27, nonresponders 22 vs. 12, facilitators 11 vs. 6, all *p* > 0.05), CPM (*females* vs *males*: inhibitors 24 vs. 20, nonresponders 26 vs. 42, facilitators 14 vs. 10, all *p* > 0.05) or offset paradigms (*females* vs *males*: inhibitors 8 vs. 4, nonresponders 11 vs. 6, facilitators 3 vs. 5, all *p* > 0.05).

### 4.2 Pain Intensity and Influences of Thermode Temperature, Pain Intensity Variability

Examination of the magnitude of pain intensity changes revealed similar inhibitor group pain decreases for placebo and CPM but significantly greater decreases for the offset compared with the placebo paradigms (mean ± SEM *placebo* vs *CPM* vs *offset* − 28.69 ± 1.78 vs. −34.84 ± 3.35 vs. −43.29 ± 6.26; *p* < 0.05 placebo<offset) (Figure 2C). While the magnitude of pain intensity changes for the nonresponder groups were similar across all three paradigms (*placebo* vs *CPM* vs *offset* 1.21 ± 1.60 vs. −1.07 ± 1.22 vs. 2.78 ± 4.37; *p* < 0.05 placebo<offset), facilitator magnitudes were greater for offset compared with CPM, but similar when comparing offset with placebo (*placebo* vs *CPM* vs *offset* 34.80 ± 4.73 vs. 26.51 ± 3.99 vs. 66.62 ± 15.82; *p* < 0.05 CPM<offset). Examination of the potential effects of sex revealed no significant differences between females and males with respect to pain intensity changes in the inhibitor, nonresponders or facilitatory groups during placebo, CPM, or offset paradigms (Table 2). Similarly, age was not significantly different between the three groups for any of the three paradigms.

**TABLE 2:** Pain intensity change, thermode temperature and pain intensity variabilities during placebo, condition pain modulation (CPM) and offset analgesia paradigms in those individuals classified as inhibitors, nonresponders or facilitators.

		% pain change (mean ± SEM)	Age (mean ± SEM)	Thermode temp (°C) (mean ± SEM)	Control scan variability (SD) (mean ± SEM)	Scan variability (SD) (mean ± SEM)
*Placebo*						
Inhibitors	All	−28.69 ± 1.78*	23.39 ± 0.52	46.84 ± 0.13	5.48 ± 0.44*	6.78 ± 0.60
	Females	−32.45 ± 3.04	23.23 ± 0.98	46.82 ± 0.19	5.89 ± 0.78	8.45 ± 1.13
	Males	−25.63 ± 1.93	23.52 ± 0.51	46.85 ± 0.18	5.14 ± 0.48	5.42 ± 0.45
Nonresponders	All	1.21 ± 1.60^ꝉ^	23.62 ± 0.81	46.63 ± 0.16	9.32 ± 0.86	7.91 ± 0.75
	Females	0.81 ± 2.09	22.41 ± 0.49	46.80 ± 0.21	9.29 ± 1.09	7.39 ± 0.83
	Males	1.95 ± 2.53	25.83 ± 2.01	46.33 ± 0.19	9.36 ± 1.47	8.87 ± 1.49
Facilitators	All	34.80 ± 4.73^#^	23.00 ± 0.88	46.82 ± 0.25	8.59 ± 1.43^#^	6.30 ± 0.79
	Females	35.53 ± 6.47	23.09 ± 1.10	46.68 ± 0.33	9.87 ± 1.99	6.57 ± 1.06
	Males	33.47 ± 7.04	22.83 ± 1.62	47.08 ± 0.40	6.23 ± 1.53	5.81 ± 1.25
*CPM*						
Inhibitors	All	−34.84 ± 3.35*	31.85 ± 2.01	46.44 ± 0.16*	8.11 ± 0.94	6.50 ± 0.43
	Females	−38.48 ± 5.04	29.42 ± 2.75	46.21 ± 0.22	8.74 ± 1.64	5.73 ± 0.52
	Males	−30.46 ± 4.13	34.77 ± 2.88	46.73 ± 0.23	7.37 ± 0.68	7.39 ± 0.69
Nonresponders	All	−1.07 ± 1.22 ^ꝉ^	32.39 ± 1.68	46.90 ± 0.12	8.98 ± 0.72	6.86 ± 0.48
	Females	−0.86 ± 2.26	31.61 ± 2.66	46.63 ± 0.17	10.18 ± 1.43	8.09 ± 0.98
	Males	−1.21 ± 1.42	32.87 ± 2.18	47.05 ± 0.16	8.19 ± 0.76	6.04 ± 0.45
Facilitators	All	26.51 ± 3.99^#^	32.29 ± 2.21	46.88 ± 0.20	7.92 ± 0.78	5.05 ± 0.59
	Females	21.00 ± 4.68	27.92 ± 1.62	46.64 ± 0.27	8.06 ± 1.07	4.22 ± 0.68
	Males	34.22 ± 6.46	38.40 ± 4.20	47.20 ± 0.29	7.74 ± 1.19	6.14 ± 0.96
*Offset*						
Inhibitors	All	−43.29 ± 6.26*	23.37 ± 0.62	46.46 ± 0.26	5.63 ± 0.94*	10.96 ± 1.38
	Females	−47.84 ± 8.10	23.75 ± 0.82	46.19 ± 0.34	6.41 ± 1.38	11.63 ± 1.94
	Males	−34.20 ± 10.95	23.50 ± 1.19	47.00 ± 0.35	4.06 ± 0.33	9.61 ± 2.00
Nonresponders	All	2.78 ± 4.37 ^ꝉ^	26.06 ± 1.92	46.71 ± 0.15	10.24 ± 1.32 ^ꝉ^	11.88 ± 0.96
	Females	2.57 ± 5.48	24.73 ± 3.59	46.82 ± 0.19	10.48 ± 1.37	12.80 ± 0.79
	Males	3.18 ± 7.42	28.50 ± 4.86	46.50 ± 0.26	9.80 ± 1.86	10.19 ± 1.07
Facilitators	All	66.62 ± 15.82^#^	25.50 ± 2.66	46.38 ± 0.13	10.38 ± 1.42^#^	12.01 ± 2.20
	Females	50.37 ± 5.16	28.00 ± 4.58	46.17 ± 0.17	11.78 ± 2.32	12.15 ± 1.75
	Males	73.12 ± 23.66	24.00 ± 3.46	46.50 ± 0.16	9.54 ± 1.88	11.92 ± 3.55

*Note:* *p* < 0.05 * inhibitors significantly different to nonresponders; ^#^inhibitors significantly different to facilitators; ^ꝉ^nonresponders significantly different to facilitators. Grey shaded boxes indicate significant differences.

We also examined the potential influence of the predetermined thermode temperature that evoked a moderate pain intensity. With the exception of CPM, where we identified responders displayed significantly reduced elected moderate temperature to nonresponders (mean ± SEM *°*C CPM responders: 46.44 ± 0.16; nonresponders: 46.89 ± 0.12; *p* = 0.03)—but not facilitatory responders—we found no significant differences in thermode temperatures between the three groups for any of the three paradigms and no influence of sex (Table 2). Furthermore, we found that pain intensity rating variability during the control scans was significantly different in placebo and offset paradigms, with significant differences between inhibitor and both nonresponder (mean ± SEM *SD control scan* placebo analgesia inhibitor: 5.48 ± 0.44; nonresponder: 9.31 ± 0.86; *p* < 0.001, offset analgesia inhibitor: 5.63 ± 0.94; nonresponder: 10.24 ± 1.32, *p* = 0.01) and facilitator groups (mean ± SEM *SD control scan* placebo analgesia inhibitor: 5.48 ± 0.44; facilitator: 8.59 ± 1.43; *p* = 0.007, offset analgesia inhibitor: 5.63 ± 0.94; facilitator: 10.38 ± 1.42, *p* = 0.01). That is, those that subsequently displayed a facilitatory response during placebo and offset scans had greater control scan pain intensity rating variability than those individuals that displayed an inhibitory response (Figure 3A). There was no significant group difference in pain rating variability during the placebo, CPM or offset scans and no significant influence of sex on pain rating variability (Table 2).

### 4.3 Psychological Measures

Single factor ANOVA and two‐sample *t*‐tests revealed neither main effects of group nor significant between‐group effects in inhibitors, nonresponders and facilitator groups for the participants in the placebo, CPM or offset paradigm groups for the STAI, PCS, BIS or BAS questionnaires (*p* > 0.05, two‐sample *t*‐test) (Table 3). However, single factor ANOVA revealed a main effect of group for LOT‐R scores in both placebo (*F*_[2,95]_ = 3.84, *p* = 0.02) and offset (*F*_[2,34]_ = 3.85, *p* = 0.03) analgesia groups. Two‐sample *t*‐tests identified that for the placebo paradigm, inhibitors recorded significantly greater LOT‐R scores compared with facilitators (mean ± SEM LOT‐R PBO *Inhibitors* 15.63 ± 0.63, *facilitators* 11.94 ± 1.06, *p* = 0.006), although not different from nonresponders (mean ± SEM LOT‐R PBO *Inhibitors* 15.63 ± 0.63, *nonresponders* 14.09 ± 0.88, *p* = 0.15). Furthermore, within the offset paradigm, inhibitors recorded significantly greater LOT‐R scores than nonresponders (mean ± SEM LOT‐R OA *Inhibitors* 17.00 ± 1.24, *nonresponders* 13.13 ± 1.20, *p* = 0.04), although not different from facilitators (mean ± SEM LOT‐R OA *Inhibitors* 17.00 ± 1.24, *facilitators* 17.25 ± 1.26, *p* = 0.89). No main effect of group on LOT‐R scores was identified within the CPM paradigm (*p* > 0.05, single factor ANOVA).

**TABLE 3:** Questionnaire values for participants in the placebo, condition pain modulation (CPM) and offset analgesia paradigms in those classified as inhibitors, nonresponders or facilitators.

	LOT‐R (mean ± SEM)	STAI‐S (mean ± SEM)	STAI‐T (mean ± SEM)	PCS (mean ± SEM)	BIS (mean ± SEM)	BAS (mean ± SEM)
*Placebo*						
Inhibitors	15.63 ± 0.63^#^	42.86 ± 1.27	36.74 ± 1.82	13.94 ± 1.49	21.02 ± 0.64	40.90 ± 0.68
Nonresponders	14.09 ± 0.89	42.65 ± 1.12	32.53 ± 1.86	12.94 ± 1.61	22.38 ± 0.56	39.71 ± 0.88
Facilitators	11.94 ± 1.10	42.77 ± 2.06	38.82 ± 2.44	15.00 ± 2.19	21.19 ± 1.09	41.53 ± 1.30
*CPM*						
Inhibitors	14.57 ± 0.93	31.51 ± 1.39	19.27 ± 1.88	7.67 ± 1.31	20.34 ± 0.85	37.10 ± 1.47
Nonresponders	15.95 ± 0.65	28.80 ± 0.97	16.01 ± 1.47	7.71 ± 0.84	19.91 ± 0.40	40.09 ± 0.59
Facilitators	12.00 ± 0.79	33.35 ± 2.24	20.59 ± 2.66	8.53 ± 2.23	22.50 ± 0.48	38.33 ± 0.68
*Offset*						
Inhibitors	17.00 ± 1.24^#^	32.50 ± 2.41	24.25 ± 2.76	11.25 ± 1.74	21.25 ± 1.30	39.42 ± 1.58
Nonresponders	13.31 ± 1.20	31.59 ± 2.34	21.94 ± 2.42	12.13 ± 1.46	22.69 ± 0.72	38.38 ± 0.97
Facilitators	17.25 ± 1.26	31.75 ± 2.42	20.25 ± 2.27	9.38 ± 3.28	19.88 ± 1.19	41.13 ± 1.32

*Note:* ^#^Inhibitors significantly different to facilitators, ^ꝉ^inhibitors significantly different to nonresponders. (uncorrected *p* < 0.05). Grey shaded boxes indicate significant differences.

## 5 Discussion

This investigation found that whilst PA, CPM and OA paradigms evoked significant decreases in pain intensity in 30%–50% of individuals, between 50% and 70% of individuals did not mount these modulatory phenomena, and importantly, of these individuals, approximately 20% displayed a significant pain intensity *increase*. This response variability raises the possibility that the neural circuitry underpinning pain decreases versus increases is underpinned by activity changes in different neural circuits. Interestingly, we also found that for both PA and OA paradigms, greater pain intensity rating variability during the initial control scan was associated with facilitatory rather than inhibitory responses. Furthermore, of numerous psychological measures, only LOT‐R scores were associated with whether an individual displayed pain increases, decreases or no change. That is, placebo facilitators had significantly lower LOT‐R scores than placebo inhibitors, with neither group differing from nonresponders, suggesting that placebo facilitators have reduced dispositional optimism compared with inhibitors.

Our results show similar proportions of individuals that display either an inhibitory, no change, or facilitatory response are consistent between the three experimental paradigms. Previous studies have shown that some individuals report pain intensity increases particularly during CPM (Firouzian et al. 2020; Graeff et al. 2021; Harper et al. 2018; Schliessbach et al. 2019), and others have reported pain increases during placebo paradigms (Aslaksen 2021; Branco et al. 2023). We report here that, like placebo and CPM, OA is also capable of evoking significant pain intensity increases in a similar proportion to that during placebo and CPM. Furthermore, we extend earlier studies to show that placebo and CPM paradigms applied to different body regions evoke similar proportions of inhibitors, nonresponders and facilitators.

It has been reported that placebo response magnitudes are negatively correlated with anxiety and fear of pain (Corsi and Colloca 2017), and although a meta‐analysis concluded that CPM magnitudes are not related to anxiety or pain fear, a secondary analysis revealed that pressure‐based CPM responses were correlated with anxiety and heat‐based CPM with depression (Nahman‐Averbuch et al. 2016). Given emerging evidence which suggests head pain is more emotionally intense and distressing than body pain (Schmidt et al. 2016), we hypothesised that placebo and CPM facilitators would have greater anxiety levels and that face placebo and CPM would evoke greater proportions of facilitators than arm placebo or CPM. We found no such relationships, with no significant differences between inhibitor, nonresponder and facilitators groups with respect to state or trait anxiety scores or any difference in the proportion of individuals that were inhibitors, nonresponders or facilitators between face compared with body placebo or CPM. We did however find that facilitators displayed slightly higher pain intensity ratings during offset compared with placebo or CPM paradigms.

We also found no significant differences in age or sex between inhibitor, nonresponder or facilitator groups for any pain modulatory paradigms. Some studies report larger CPM analgesic effects in males than females (Arendt‐Nielsen et al. 2008; Staud et al. 2003) and in younger compared to older participants (Lariviere et al. 2007; Riley 3rd et al. 2010), although a recent study has reported that sex and age factors very little in the variance of CPM expression (Graeff et al. 2021). Similarly, the effects of sex on PA appear inconsistent, with some studies reporting female, some male and some no sex interactions with placebo responsivity (Enck and Klosterhalfen 2019). Moreover, PA expression appears preserved in the elderly (Wrobel et al. 2016). While far fewer studies have explored OA, a review suggested that there is little evidence for sex differences in responses, although there are indications that offset‐ability reduces with age (Hermans et al. 2016). Our study extends these previous studies by also exploring the influence of sex and age on facilitatory responses, and we find no significant effect of age or sex on the direction of pain change during any of the three experimental paradigms. It should be noted that our age distributions were relatively restricted to younger adults, and thus future studies exploring the effects of age on pain modulatory direction are needed.

Whilst we found no significant effects of pain catastrophising, state and trait anxiety, and behavioural inhibition/activation. These lack of associations are consistent with a previous meta‐analysis which revealed no significant relationships between CPM responses and anxiety, depression and pain catastrophising (Nahman‐Averbuch et al. 2016). Similarly, a recent systematic review and meta‐analysis concluded that there is no consistent evidence that behavioural inhibition and anxiety influence placebo responsivity (Kang et al. 2023). Whilst this recent study also reported no consistent significant relationship between placebo responsivity and optimism (Kang et al. 2023), we did find that placebo inhibitors displayed significantly higher LOT‐R scores, that is, greater optimism, than facilitators. This result is however similar to a recent systematic review which concluded that optimism was relatively consistently associated with increased placebo responses (Kern et al. 2020). Our finding might reflect our grouping of participants into inhibitors, nonresponders and facilitators, since we found no significant differences between nonresponders and either inhibitors or facilitators and thus, we essentially assessed two ends of the pain modulation range. It is interesting to note that whilst optimism was related to placebo responses, it was not related to either CPM or OA, suggesting that optimism can affect pain modulatory circuitry associated with placebo but not CPM or OA.

Interestingly, we found that both placebo and CPM responses were associated with pain intensity rating variability during the control scans. It has been previously shown that individuals that rate the intensity of a noxious stimulus with more accuracy, that is, precision, display more pronounced PA effects (Grahl et al. 2018). Our results are consistent with this previous result in that individuals that showed less pain intensity rating variability during the control scan were more likely to report a subsequent greater PA and OA. We have previously shown that pain intensity rating variability is associated with functional magnetic resonance imaging (fMRI) signal changes in the dorsolateral prefrontal cortex (dlPFC) (Crawford, Mills, et al. 2023) and dlPFC connectivity with the midbrain periaqueductal grey (PAG) (Meylakh et al. 2024). The dlPFC is considered to be an interface between cognitive processing and pain modulation (Seminowicz and Davis 2006; Seminowicz and Moayedi 2017) and has been directly linked to placebo and CPM analgesic ability (Crawford, Meylakh, et al. 2023; Crawford, Mills, et al. 2023; Krummenacher et al. 2010; Youssef et al. 2016a). The dlPFC‐PAG circuitry may be altered in those less proficient in monitoring their pain in response to consistent moderate intensity stimuli and may also be responsible for driving pain modulatory responses during placebo and CPM paradigms. Why pain intensity variability does not appear to influence CPM responsiveness is unknown; however, it implies that placebo and offset analgesia may more heavily rely on cognitive and sensory integration than CPM.

Indeed, it was recently proposed that PA can be predicted, modelled and encoded under a Bayesian framework (Büchel et al. 2014; Pagnini et al. 2023). In this model, conditioning cues or expectations of pain relief (likelihood) interact with unconditioned pain sensitivity (prior) to form the posterior of pain relief during PA intervention. The difference between the likelihood and prior is the prediction error differential (Schenk et al. 2017). Since a similar proportion of individuals respond to CPM, OA and PA, we propose that CPM and OA may also be encoded under similar Bayesian systems. We suggest that CPM is generated as some function of noxious sensitivity, likely to both the conditioning and test stimuli, which together trigger descending modulation through medullary gating of ascending nociceptive input. In our hands, OA shared similar traits to PA; that is, reduced pain intensity variability in the control scan encoded greater OA responses. Since the width of Bayesian curves is a product of the variance of data entered, likely offset also functions under some conceivable Bayesian model, with a more precise prior—control scan variability—being key to mounting offset analgesia of greater magnitude.

It is important to note some limitations. First, it was not possible to counterbalance the design such that the control scan was always stimulated first and the placebo, CPM or offset second. This ordering effect could have introduced sensitisation and or habituation effects although this is unlikely given the variability in responses. Second, we had fewer participants undergo the offset protocol and only applied this protocol to the arm and not also the face, as was the case for placebo and CPM. While this meant that offset results were less powered than the other paradigms, the finding that all three paradigms resulted in similar proportions of inhibitors, nonresponders and facilitators suggests that the reduced offset numbers were unlikely to affect these results. It is also important to note that each response class: facilitator, nonresponder and inhibitor are not entirely distinct when considering an absolute value of ‘pain intensity change’ between scans, since a permutation model depends on the variability of within‐stimulus pain ratings for generating sample distribution curves. That is, we cannot designate a cut‐off value of percentage pain change to classify every individual as either of these three response classes, and rather whether a participant falls into any single class depends on their degree of variability encoding to repeated identical noxious stimuli. To our knowledge, no clear consensus exists on whether a categorical or continuous approach should be taken to classifying endogenous pain modulatory phenomena, however, what we describe here demonstrates a clear trimodal distribution that presents when these phenomena are investigated at high power, such that either a large increase, no change or a reduction in perceived pain is observed, with largely similar proportions of individuals being assigned one of these three classifications between‐phenomena. Finally, we collected pain rating data consistently throughout the scanning sequence, requiring participants to both perceive and accurately report their pain at all times. This procedure reduces the likelihood of series position effects or response biases associated with collecting pain ratings after a series of stimuli (Hrobjartsson et al. 2011), although it may have in part contributed to phenomena of pain rating variability.

## Author Contributions

**Lewis S. Crawford:** conception and design, data acquisition, analysis, data interpretation, article draft and final approval; **Ashleigh Wake:** data acquisition, article draft and final approval; **Rebecca V. Robertson:** data acquisition, article draft and final approval; **Allan Peng:** data acquisition, article draft and final approval; **Noemi Meylakh:** data acquisition, article draft and final approval; **Damien C. Boorman:** data acquisition, article draft and final approval; **Leana Sattarov:** data acquisition, article draft and final approval; **Alister Ramachandran:** data acquisition, article draft and final approval; **Vaughan G. Macefield:** data acquisition, article draft and final approval; **Kevin A. Keay:** funding acquisition, conception and design, article draft and final approval; **Luke A. Henderson:** funding acquisition, conception and design, data interpretation, article draft and final approval, integrity of the work as a whole, from inception to published article.

## Disclosure

Originality: This work is the original work of the authors and is not under consideration at any other journal.

## Supporting information


Appendix S1.

## Data Availability

Anonymised data files may be made available to qualified investigators upon request.
