# Supplementary material for: Conditioned Pain Modulation, Placebo and Offset Analgesia: Rates of Behavioural Expression of Inhibitory, Nonresponse and Facilitatory Pain Modulatory Effects
Source: Eur J Pain. 2025 Jul 22;29(7):e70088. doi: 10.1002/ejp.70088 (PMC12281077; doi:10.1002/ejp.70088)
Supplement: Supplementary file 1 — Appendix S1. [file EJP-29-0-s001.docx]

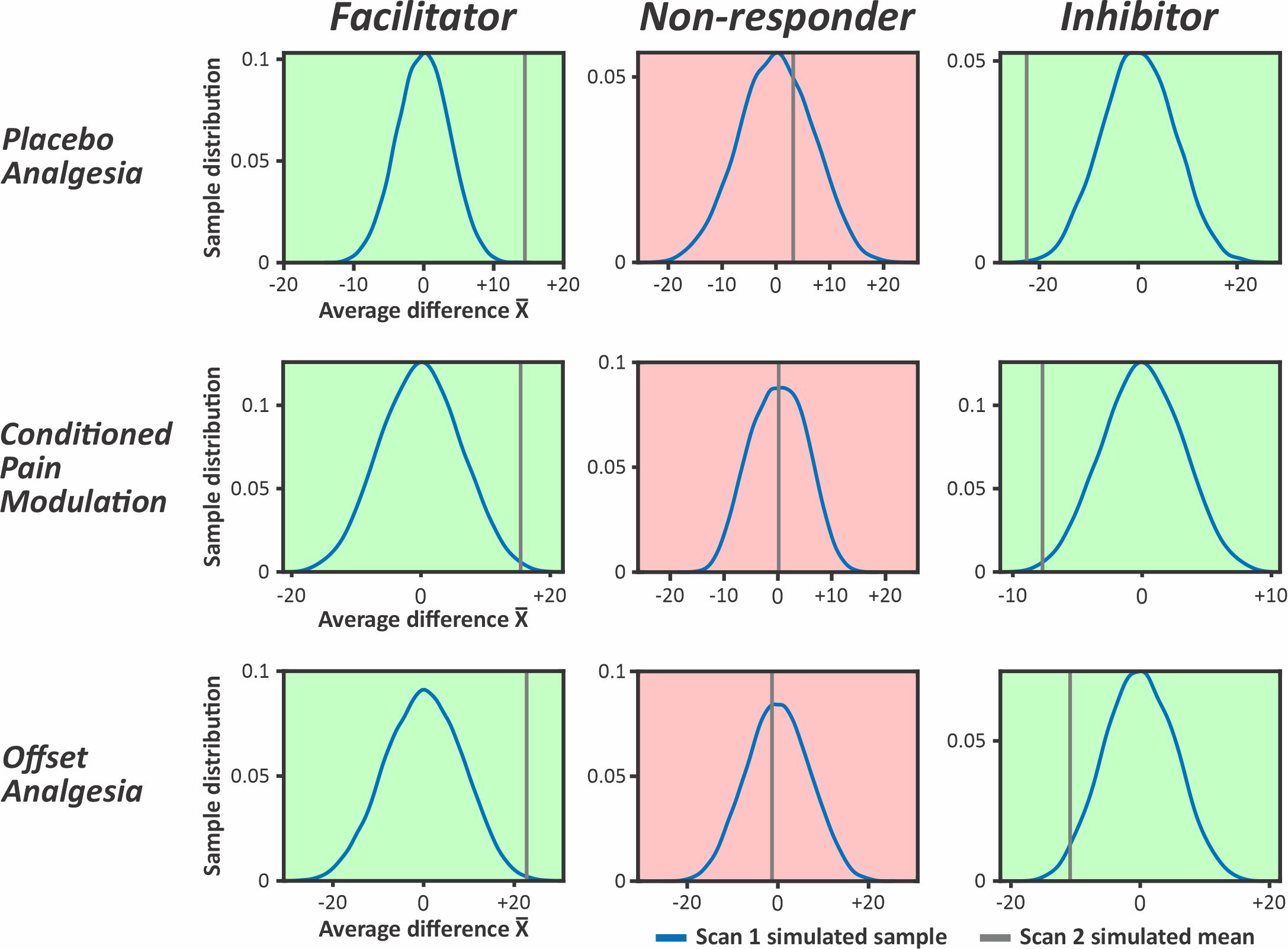


**Supplementary Figure 1. Example bootstrapped permutation output for delineating facilitator, non-responder, and inhibitor groups.** By first entering rating values to each noxious stimuli of the control scan for each phenomenon and generating a distribution curve of 10,000 artificial samples, the same process was repeated for either the placebo, conditioned pain modulation, or offset analgesia scan with significance determined between the two artificial samples through a one-tailed t-test. Visualizing these outputs shows an example facilitator with mean pain intensity in scan two – that is, either the placebo, conditioned pain modulation, or offset analgesia scan (gray line) – as significantly higher than the average simulated sample of the relevant control scan (blue line). In non-responders, scan two simulated average falls similarly to the simulated distribution of pain responses to the control scan, and in inhibitors the scan two simulated average falls significantly lower than the relevant control scan stimulated distribution.

Supplementary Table 1. Administered psychological questionnaires, their measured behavioural construct, lower and upper scoring boundaries, and clinically relevant scoring brackets.

| Questionnaire | Measured Construct | Lower : Upper Boundary | Scoring brackets |
| --- | --- | --- | --- |
| Revised Life Orientation Test (LOT-R) | Trait Optimism – General expectation that good things will happen | 0 (lower)  24 (upper) | **NA** – greater scores indicate greater dispositional optimism |
| State-Trait Anxiety Inventory – State (STAI-S) | State Anxiety – temporary shifts in emotional state toward tension and apprehension | 20 (lower)  80 (upper) | 20-37 no or low anxiety  38-44 moderate anxiety  45-80 high anxiety |
| State-Trait Anxiety Inventory – Trait (STAI-T) | Trait Anxiety – general tendency to perceive threat and respond anxiously | 20 (lower)  80 (upper) | 20-28 no anxiety  29-37 low anxiety  38-44 moderate anxiety  45-80 high anxiety |
| Pain Catastrophizing Scale (PCS) | Behavioural responses to pain - Rumination, Magnification, Helplessness | 0 (lower)  52 (upper) | 0 – 29 clinically irrelevant  30 – 52 significant pain catastrophizing |
| Behavioural Activation Scale (BAS) | Approach behaviours – drive, reward, and fun-seeking | 13 (lower)  52 (upper) | **NA** – greater scores indicate more approach behaviours are present |
| Behavioural Inhibition Scale (BIS) | Avoidance behaviours – likelihood to experience negative emotions in perceived threat / conflict | 5 (lower)  20 (upper) | **NA** – greater scores indicate more avoidance behaviours are present |
